# Paradoxes of Hymenoptera flight muscles, extreme machines

**DOI:** 10.1007/s12551-022-00937-7

**Published:** 2022-02-23

**Authors:** Tony Hickey, Jules Devaux, Vijay Rajagopal, Amelia Power, David Crossman

**Affiliations:** 1 School of Biological Sciences, University of Auckland, Auckland, New Zealand; 2 Department of Physiology, University of Otago, Dunedin, New Zealand; 3 Department of Biomedical Engineering, University of Melbourne, Melbourne, VIC Australia; 4 Faculty of Medical and Health Sciences, University of Auckland, Auckland, New Zealand

**Keywords:** Bees, Ants, Extreme contraction rate, Mitochondria, Creatine kinase, Adenylate diffusion

## Abstract

In the Carboniferous, insects evolved flight. Intense selection drove for high performance and approximately 100 million years later, Hymenoptera (bees, wasps and ants) emerged. Some species had proportionately small wings, with apparently impossible aerodynamic challenges including a need for high frequency flight muscles (FMs), powered exclusively off aerobic pathways and resulting in extreme aerobic capacities. Modern insect FMs are the most refined and form large dense blocks that occupy 90% of the thorax. These can beat wings at 200 to 230 Hz, more than double that achieved by standard neuromuscular systems. To do so, rapid repolarisation was circumvented through evolution of asynchronous stimulation, stretch activation, elastic recoil and a paradoxically slow Ca^2+^ reuptake. While the latter conserves ATP, considerable ATP is demanded at the myofibrils. FMs have diminished sarcoplasmic volumes, and ATP is produced solely by mitochondria, which pack myocytes to maximal limits and have very dense cristae. Gaseous oxygen is supplied directly to mitochondria. While FMs appear to be optimised for function, several unusual paradoxes remain. FMs lack any significant equivalent to the creatine kinase shuttle, and myofibrils are twice as wide as those of within cardiomyocytes. The mitochondrial electron transport systems also release large amounts of reactive oxygen species (ROS) and respiratory complexes do not appear to be present at any exceptional level. Given that the loss of the creatine kinase shuttle and elevated ROS impairs heart function, we question how do FM shuttle adenylates at high rates and tolerate oxidative stress conditions that occur in diseased hearts?

## Evolution of flight

Hymenoptera (bees, wasps, hornets, sawflies and ants) compete with Coleoptera (beetles) as the most speciose order on Earth (Forbes et al. 2018). While there are over 150,000 nominal hymenopteran species, a potential 1 million undescribed hymenopterans are thought to remain among parasitic wasps. This group inhabits environments spanning from cold temperate habitats to hot deserts. Indeed, the most thermotolerant animal species the Saharan ant (*Cataglyphis bombycine*) exists at 47 °C and tolerates ambient temperatures up to 70 °C, and can travel at 108 body lengths per second (for entertainment’s sake that equates to 200 m s^−1^, or 720 km h^−1^ for a 1.80 m human) (Wehner et al. 1992). While this must require phenomenal muscle contractile capacities (in part promoted by high temperatures), perhaps more remarkable are the contractile properties of flight muscles in bees and wasps. Here we review unusual properties of flight muscles (FM) of bees and wasps. We discuss how adaptations to support extreme rates of FM contraction have evolved, yet paradoxically how they done so by breaking rules that appear to impede muscle contraction and associate with heart failure in vertebrates.

## Insect flight differs from birds

Flight is governed by aerodynamic properties of air, such as viscosity and density, and these interact with drag and wing size span and form components of the Reynolds equation (*Re* = *Vc*/v, where *V* is flight, or wing speed, *c* is the chord length, and *ν* is the kinematic viscosity of the fluid in which the aerofoil operates). These factors determine how flight can be achieved, as they dictate how wings can develop forces. As flying insects are typically small, flight contrasts to that using the aerofoiled wings of larger birds, which have been honed to increase efficiency. Although both result in lift, a bird wing (excluding hummingbirds) attacks the air at a gentle angle of attack, in part directing air downwards and also partitioning the air to generate pressure differentials (Dickinson 2006). Bird wings mostly flap up and down, and aim to reduce drag resulting from turbulence and vortex formation. In contrast, insect wings, such as those of flies, wasps or beetles, flap back and forth, and the wings leading edges attack the air at extreme angles (Dickinson 2006). This deliberately forms vortices, and these are maintained and sum to generate lift. This approach requires high frequency oscillations, with rapid wing accelerations, and consequently, an inefficient process is harnessed to generate lift (Dickinson 2006).

## Birds and the bees

Hummingbirds contract muscles at up to 90 Hz, a frequency that appears to be at the upper limit for force generation in vertebrates (Rome and Lindstedt 1998). Honeybee, bumblebee and wasp FMs contract at rates of 200 to 230 Hz, and midges approach 1000 Hz (Vishnudas and Vigoreaux 2006). Members of the group Hymenoptera, specifically wasps and bees, also fly with payloads, lifting more than their body mass, with wasps lifting prey, or prey pieces of up to 150% of their body mass (Nalepa and Swink 2018). Bumblebees (*Bombus* sp.) fly with disproportionately small wings and were once thought to defy gravity. Yet, bumblebees have significant excess flight capacities as they can fly at atmospheric densities and O_2_ pressures lower than at Mount Everest’s summit (~ 9000 m, less than 1/3 of the air density at sea level) (Altshuler et al. 2005). Although the biomechanics of bumblebee wing movement have since been resolved (Dudley and Ellington 1990), there are several other similar paradoxes, and parallels can be drawn between FM and vertebrate striated muscles (Hodge 1956) with a closer resemblance to cardiac muscle (Bullard and Pastore 2019). However, clearly FM still differs substantially and raises questions, such as how do FMs support high activities with a near absence of lactate dehydrogenase? Perhaps the most intriguing FM functions without an equivalent to the ADP/ATP shuttle, such as the creatine or arginine kinase system. How do they deal with enormous ATP demands and issues of adenylate diffusion?

## Modern insect flight muscles

Here we discuss modern insect FM and crudely differentiate modern versus ancient volant insects. Ancient species such as dragonflies (*Anisoptera*) emerged in the Carboniferous (325 Mya), and modern insects such as Hymenopterans emerged in the Triassic (250 Mya), with eusociality appearing in the Cretaceous (146–66 Mya) (Peters et al. 2017). The group likely evolved an ectophytophagus species, with an ability to shift between haploidy and diploidy.

**Fig. 1:**
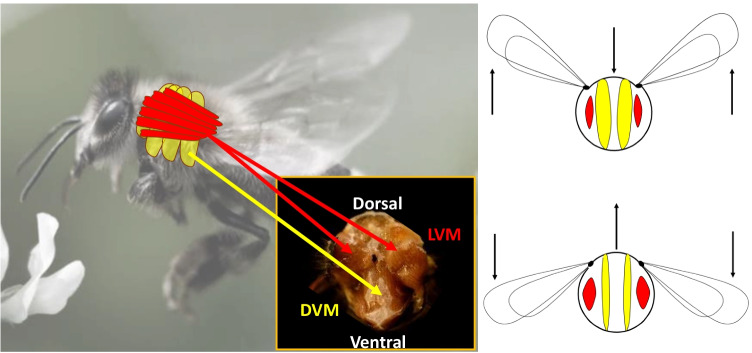
The flight muscles (FM) of Hymenoptera occupy at least 90% of the volume of the thorax and consist of two main large motor units, which consist of dorsal–ventral muscles (DVM) and lateral-ventral muscles (LVM). Additional smaller muscle sets are not shown, and these aid wing control and initiate stretch activation of the large motor units. Note that the FMs do not directly attach to wings, instead they attach to the interior thorax wall. The wings are attached to the mesothorax via the tegula, and these are moved up and down as they are pivoted around the static sides of the thorax, while a rigid plate attached by an elastic cuticle hinge allows flexion. The DVM contract to pull down on the rigid upper plate and LVM contract to force raise the upper plate. The stretching of opposing muscle sets further generates stretch activation, and the muscle sets act in resonance, until calcium is removed from the FM cytosol by a slow acting SR

FMs of dragonflies resemble vertebrates in that they attach more directly to wing structures. Also, for every contraction and wingbeat, there is a coincident/synchronous neuronal stimulation (synchronous FM). In contrast, in modern insects, neuronal stimulation is asynchronous, in that there are > 10 wingbeats/contractions per depolarisation event. Modern asynchronous insect FMs are attached to the interior walls of the thorax exoskeleton, which is effectively a hollow ball with rigid sections and highly compliant, elastic sections of cuticle (Fig. 1). These allow thorax deformation and the wings are attached in such a manner that they are levered off the exoskeleton. Therefore, as the thorax deforms, the wings pivot on lever arms to amplify FM shortening and generate high velocities at wingtips. The main power-generating FMs within the thorax consist of two groups that run dorso-ventral and dorso-longitudinal to the thorax and oppose each other and contract alternately (Dickinson 2006).

How modern insect FMs generate force with such high contraction frequencies is surprising. Power generation is limited by space constraints within myocytes. Force generated by contractile actomyosin motor units is typically activated and arrested through motor neuron mediated calcium (Ca^2+^) release, followed by active Ca^2+^ sequestration by the sarcoplasmic reticulum (SR). For muscle in general, both contraction and Ca^2+^ uptake require significant ATP, and for sustained efforts, this is met by mitochondrial oxidative phosphorylation (OXP). ATP stores in insect FM support less than a second of flight (Wegener 1996), and anaerobic pathways are negligible in insect FMs (see below). Consequently, mitochondria occupy 42–45% of myocyte volume, the upper limit for useful force generation, which coincidentally this is similar to that within rodent cardiomyocytes (Rome and Lindstedt 1998). In most muscles, including insect non-FM, repeated contraction cycles require rapid Ca^2+^ reuptake. FMs break from this rule.

In synchronous muscles, Ca^2+^ reuptake is slower than release, and increases in contraction frequency require the expansion of the SR. However, SR expansion diminishes force generation (Rome and Lindstedt 1998). Paradoxically, the SR volume in modern insect FMs is minimised, and cytosolic [Ca^2+^] is “poorly” managed. Motor neurons stimulate FM in a continuous yet varied tone, and this increases or decreases cytosolic [Ca^2+^] from one state to another. FM neural stimulation is asynchronous, in that one stimulation coincides with 10 or more wing beats. Ca^2+^ release still initiates contractions, yet these are limited in length, where they reach a maximum and actin and myosin cross-bridge cycling breaks down and fibrils rapidly lengthen through FM elastic recoil. This stretch occurs through tension generated by (1) a titin-like protein called flightin, (2) resistive forces imparted by the exoskeleton and (3) contraction of opposing muscle units. On lengthening, cross-bridging then resumes and the cycle repeats until Ca^2+^ is removed by a low volume, slow acting SR. This decreases ATP demand substantially, as nearly all ATP is hydrolysed by myosin heavy chain and less so than by ion pumps (Vishnudas and Vigoreaux 2006, 2007).

FM can also be separated into different units, large “dumb” powerful motors and “smarter” control units, which are also more densely innervated. When the smaller smarter units activate, their contraction opposes and stretch activates the larger powerful units, thereby these larger units require less innervation. Stretch activation also occurs in cardiac muscle, and this contraction/relaxation effectively permits FM/motor units to bounce with minimal neural stimulation, further conserving ATP (Dickinson 2006).

## Flight requires high glycolytic flux but is absolutely restricted to aerobic respiration

Aerobic capacities of insect flight muscles are extreme. The power output of a honeybee in flight under load is approximately 1 kW kg^−1^ (Feuerbacher et al. 2003), which equates to 80 kW for an average sized male human. While the mean absolute maximal anaerobic power outputs for humans approaches 1.2 kW (Davies and Sandstrom 1989), sustained aerobic power outputs approximate 450 to 480 W (6.70–7.02 W kg^−1^) (Pinot and Grappe 2014). Although metabolism scales allometrically, honeybees show a 143-fold greater power output. Note we compare aerobic power, as bees and wasps sustain flight through strictly aerobic pathways. They lack significant lactate dehydrogenase (LDH) expression and activity (Suarez 1998). Presumably this results from the considerable energetic inefficiency if anaerobic metabolism was engaged, with only 2 ATP derived per glucose compared to 32 to 38 ATP harnessing oxidative phosphorylation (Suarez 1998).

## Glycolytic flux runs at high maximal capacities

Flight is sustained on glucose and to a lesser extent proline oxidation (Suarez 2000; Suarez et al. 1996). Thus, honeybee flight muscle also appears to operate near the maximal flux capacity of non-equilibrium glycolytic enzymes. Hexokinase (HK) and phosphofructokinase (PFK) operate at almost 100% and 70% of Vmax, while mammals operate at 28% and 44% respectively (Suarez 2000). This enables FMs to function at high metabolic rates without substantial increases in enzyme concentration (Suarez 2000), as again cytosolic space allocation for glycolytic enzymes is at a premium (Suarez 1998).

Not only is the maximal capacity of Hymenoptera species remarkable, but also metabolic flux can increase and decrease 43-fold from resting to flight, and then back to resting states, with minimal change in metabolic intermediate concentrations (Sacktor, 1970). Within mammals, glycolytic control is considered to be distributed across non-equilibrium enzymes, and PFK and pyruvate kinase (PK) appear to be bound to fibrils in Drosophila (Stephan et al. 1986). As both enzymes are allosterically regulated, these may provide direct linkage with contractile machinery ATP demands to metabolic ATP supply. While HK is known to associate with mitochondrial membranes (Wallace 2005), less is known how glycolysis is regulated in FM.

## Cytosolic ATP supply and redox management in the absence of LDH?

The lack of adequate LDH in FM presents an issue. Within cells of most animals, LDH or the malate aspartate shuttle generally maintains the cytosolic NAD^+^/NADH redox state and thereby sustains glycolytic flux. While lactate may be oxidised by mitochondria in striated muscles (Kane 2014), without LDH (Crabtree and Newsholme 1972, 1975), FM glycolysis must dispose of pyruvate. Glycolytic enzyme complexes have been found bound to myofibrils (Stephan et al. 1986). However, pyruvate is acidic at physiological pH and its accumulation combined with a depletion of NAD^+^ will arrest glycolysis.

To maintain the cytosolic redox state, Hymenoptera and Dipteran FMs use the glycerol 3-phosphate (G3P) shunt (Fig. 3) (Vishnudas and Vigoreaux 2006; Sullivan et al. 2003). Cytosolic and mitochondrial G3P dehydrogenases (cG3PDH and mG3PDH) reduce glycolytic derived dihydroxyacetone phosphate (DHAP) to regenerate NAD^+^ and form glycerol 3-phosphate (G3P). G3P is then oxidised on the cytosolic side of the inner mitochondrial membrane by FAD-linked mG3PDH. This pathway differs from the slower more efficient malate aspartate shuttle, in that the G3P shunt uses fewer enzymes and intermediates, so it is much more rapid. However, this speed comes at a theoretical 40% loss of ATP that would be generated by the malate aspartate shuttle. The G3P shunt appears to be involved in tissue warming in bumblebees (Masson et al. 2017) and also, it releases significant amounts of reactive oxygen species (ROS) (Hedges et al. 2019). Overall, inefficient FMs use a less efficient cytosolic redox management system that likely involves significant oxidative stress. How these are managed are yet to be explored.

Overall, the G3P shunt supports glycolytic ATP formation and oxidative phosphorylation systems (OXPS) (Vishnudas and Vigoreaux 2006). We have recently found that the G3P shunt contributes up to 34% of total O_2_ flux in bumblebee FM (Masson et al. 2017). The binding of cG3PDH to FM myofibrils of *Drosophila* is essential for flight (Wojtas et al. 1997; Vishnudas and Vigoreaux 2006). While myofibril-bound glycolytic enzymes should in part support cytosolic myofibrillar ATP demands, only ~ 5% of the ATP formed from glucose oxidation is born at this step. Thus, enzyme attachment likely promotes communication to mediate ATP demands, but likely does not itself substantively support ATP demands. This leads to a greater issue in that there is an apparent absence of an ADP/ATP shuttle between myofibrils and mitochondria (Vishnudas and Vigoreaux 2006).

## There is no apparent adenylate shuttle in FMs

**Fig. 2:**
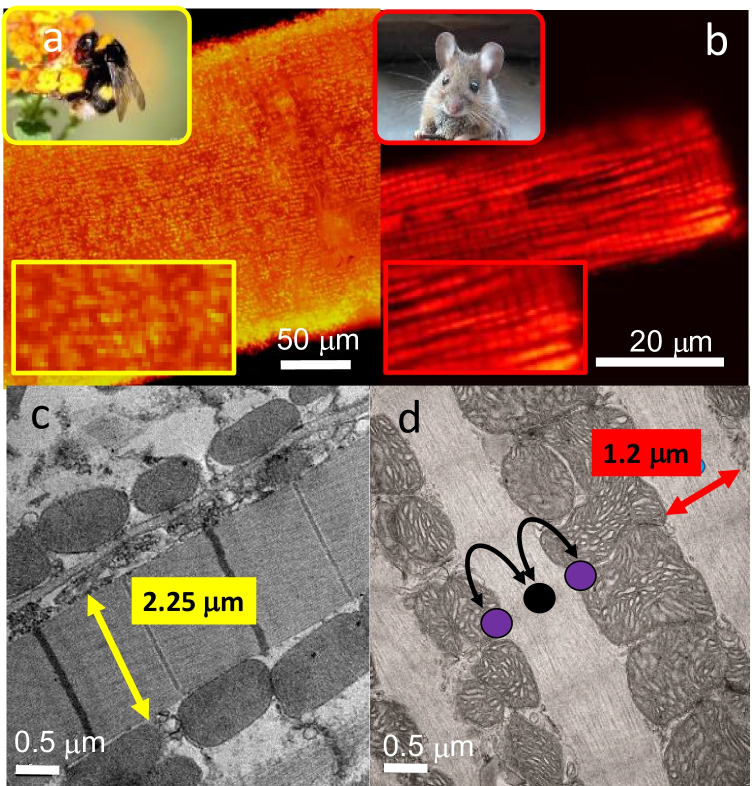
A bumblebee (*Bombus terrestris*) FM fibre and rodent cardiomyocyte respectively stained with fluorescent mitochondrial membrane potential indicators JC1 and TMRM. While the fine rows of mitochondria are apparent in both muscle types, it is worth noting the differences in magnification of each preparation (for the insets, the white bar represents 15 µm for both species). The bumblebee has significantly wider fibres, yet more punctate mitochondria. At higher magnification, additional structural differences are apparent. The z bands are highly defined in bumblebee FM and the cristae more tightly packed. Within the cardiomyocyte, cristae are fewer and less densely packed, while the distances across fibrils are less than half that observed in FM. Moreover, FM lacks mtCK (purple dots) and MMCK (black dot), which enhances energy transfer of phosphagens across the fibril

FMs lack any apparent adenylate transfer system. Creatine kinase (CK) and the analogue arginine kinase (ArgK) are absent in FMs (Newsholme et al. 1978; Wolschin and Amdam 2007; Beis and Newsholme 1975). In vertebrates, cardiac CK isoforms form an ATP shuttle system, where they effectively transport the energy within ATP from mitochondria to myofibrils via a mitochondrial CK (*mt*CK) and myofibril CK isoforms (MMCK, Fig. 2). While creatine kinase (CK) knockdown models generally show this system is not essential in mammals, muscle function is compromised at high capacities (Nahrendorf et al. 2005), muscle wastage is apparent and chronic exercise impaired (Momken et al. 2005); moreover, CK depression is observed in severe cardiomyopathies (Fowler et al. 2015).

Depressed CK also coincides with elevated ROS production (Ventura-Clapier et al. 2004; Neubauer et al. 1997; Neubauer 2007; Jullig et al. 2010); as impaired ADP delivery to mitochondria can elevate membrane potentials, this can induce reverse electron flow and promote ROS production (Murphy 2009; Chouchani et al. 2014). We have also shown that pathological depression of CK not only impairs mitochondrial ADP transport between mitochondrial and myofibrils, but also associates with elevated ROS production in failing rat hearts (Power et al. 2016). Even in permeabilised fibres from healthy hearts, the addition of creatine decreases ROS production (Power et al. 2016).

During periods of intense activity, CK shuttles support myofibrillar ATP supply (Weiss et al. 2005) and surmount the slow diffusion rates of large, negatively charged ATP and ADP molecules, to and from sites of high ATP turnover (Schlattner et al. 2006). While FMs sustain rapid ATP turnover rates (Suarez et al. 1996), at subcellular levels, ATP and in particular ADP diffuse slowly (Kekenes-Huskey et al. 2013). Creatine phosphate (CrP) and creatine diffuse further than ATP (diffusion lifetimes 57, 37 and 22 µm s^−1^ respectively), and ADP diffusion presents a significant impediment as it diffuses 12 times slower than ATP (diffusion lifetime 1.8 µm s^−1^) (Hubley et al. 1995). Addition of exogenous creatine also decreases the mitochondrial *K*_*m*_ for ADP within permeabilised cardiac muscle approximately sevenfold, and this likely results from activation of mtCK, thereby decreasing the need for ADP to diffuse across the myocyte (Saks et al. 1991). As with vertebrate myosins, accumulating ADP inhibits FM myosin and promotes rigor (Vishnudas and Vigoreaux 2006). In vertebrate myocytes, the loss of the CK shuttle increases ADP gradients across fibrils (175–225 µM/µm) and this should impede contraction (Kekenes-Huskey et al. 2013). While invertebrates have alternative phosphagen transfer systems and insects harness the arginine kinase system, arginine kinase expression is very low in FMs (Wegener 1996).

While it has been postulated that an abundance of mitochondria regimentally surrounds myofibrils and counteracts issues of ADP diffusion (Wegener 1996), however, myofibrils are ~ 2.5-fold wider than those within rodent cardiomyocytes which increases diffusion distances to the core of myofibrils (Fig. 2) (Saktor 1970). A centralised accumulation of ADP within myofibrils lacking any analogy to the CK system should result in uneven contraction and enhance ROS release from mitochondria. How can high-performance FMs circumvent apparent barriers to adenylate diffusion and elevated ROS?

## Oxygen delivery and paradoxes of electron transport system capacities

While myocyte ultrastructure of FMs have been paralleled with appearances to mammalian cardiac muscles (Vishnudas and Vigoreaux 2006; Dickinson et al. 2005), they differ substantially. FMs are absolutely dependent on oxidative phosphorylation (OXP) (Saktor 1970; Beenakkers 1984) and there are no respiratory pigments such as myoglobin (Vishnudas and Vigoreaux 2006; Saktor 1970). Paradoxically, FMs have a wider fibre diameter, or cross-sectional area, that should increase force generation (Vishnudas and Vigoreaux 2006), but it also increases substrate and oxygen diffusion distances. To circumvent diffusional barriers of oxygen, insects have evolved highly branched tracheal systems that directly interface with mitochondria to exchange gases directly (Vishnudas and Vigoreaux 2006). As FMs are likely less reliant on blood perfusion for O_2_, bee and wasp FMs have evolved a 5 ~ tenfold greater cell diameter, and ~ 2.5-fold increase in myofibril diameter (Fig. 2) (Saktor 1970).

The VO_2_ max of honeybees is phenomenal and has been measured at 1700–2000 ml (min kg)^−1^ (Suarez et al. 2005, 1996). For perspective, the typical VO_2_ max for a human is 40 ml (min kg)^−1^ (Suarez 1998) and the highest recorded is 99 ml (min kg)^−1^. On conversion to units we routinely use in the laboratory for mitochondrial function of permeabilised muscle fibres, for a bee in flight, we estimate O_2_ flues of 1240–1500 pmol (s. mg)^−1^. Flight muscles occupy approximately 90% of the thorax (Fig. 1), and the thorax accounts for 30% of body mass, and approximately 90% of the O_2_ used in flight supports FMs (Suarez 2000). Therefore, we can approximate FM O_2_ uptake in the animal, and it should equate to 4135–5000 pmol O_2_ (s mg)^−1^ (the VO_2_ max of the thorax should approximate 5,500–6500 ml (min kg)^−1^!).

We measured oxygen flux of permeabilised FM fibres from honeybee, bumblebee (*B. terestris*) and German wasp (*V. germanicus*) at 35 °C and supersaturating oxygen concentrations, O_2_ flux in OXP supported by complex I, II and G3PDH approaches 800 pmol O_2_ (s mg)^−1^, and the flux through cytochrome c oxidase using TMPD ascorbate approaches 2300 pmol O_2_ (s mg)^−1^. These measures are double those for rodents, and some reported values for honeybee FM using the same equipment (Syromyatnikov et al. 2019). However, our measurements remain substantially lower than estimates of O_2_ flux in vivo.

The disparity between in vitro and in vivo mitochondrial O_2_ fluxes are contrary to what is found in mammalian muscles, where mitochondrial O_2_ flux in vitro exceeds intact tissue O_2_ flux (Boushel et al. 2011). The shortfall in mitochondrial capacity may result from omission of substrates, i.e. proline was omitted. However, the addition of proline does not double or quintuple O_2_ flux (Syromyatnikov et al. 2019). While the state of preparations may have damaged mitochondria, typical measures of quality were excellent and the P/O ratios in bumblebee (*B terrestris*) FM assayed in equivalent settings exceeded 3, indicating high coupling efficiencies using pyruvate (Masson et al. 2017).

**Fig. 3:**
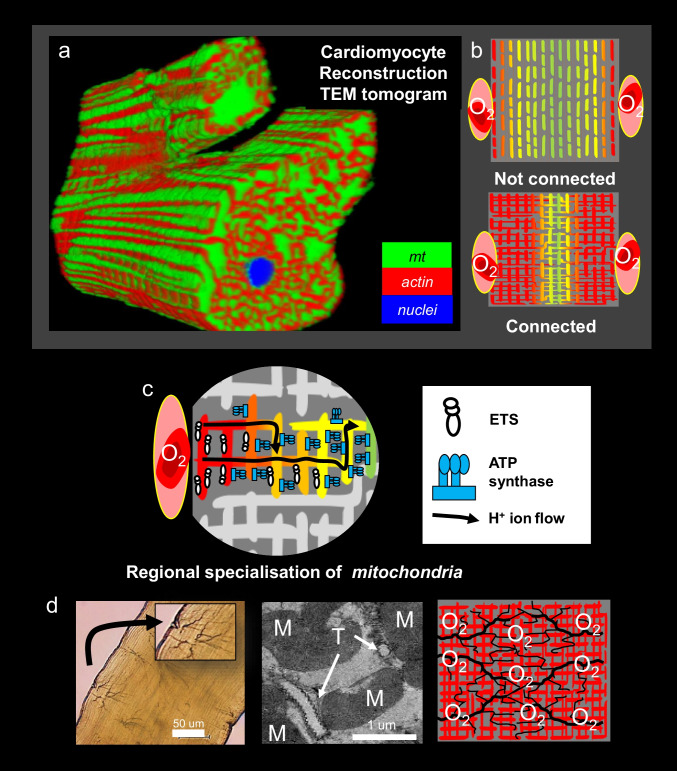
Reconstruction of serial block-face transmission electron micrographs images (**a**) shows that mitochondria are in intimate contact and form networks that extend throughout a rat cardiomyocyte. Mitochondria within cardiomyocytes may not act as punctate distinct organelles (**b**, upper), but function as interconnected networks (**b**, lower), putatively to better harness elevated O_2_ in blood by capillaries to the plasma membrane. Possibly enhances membrane potential formation (red) deep within cardiomyocytes, where elevating **c** electron transport system units (ETS) of mitochondria proximal to the plasma membrane occurs where O_2_ is richest. Through ion transfer among networks, mitochondrial polarisation is enhanced deeper within cells, where phosphorylation system components (ATP synthase) are elevated. Overall, this aids ATP synthesis where O_2_ is limited. **d** Insect FMs have much thicker, more regimented organisation of fibrils, and are intubated by tracheole systems (**d**, left) that deliver gas directly into FMs. Given that tracheoles directly contact mitochondria (centre, T), we question how does this affect the capacity and arrangement of mitochondria, i.e., what is their respiration capacity in a gas phase, are they interconnected, and how is hyperoxia managed?

Another possibility is that O_2_ may be limiting. While we used supersaturating O_2_, FM fibres were tested in respiration media. FMs in vivo are directly supported by atmospheric gases. Tracheole branches are small (40–200 nm diameter) and penetrate sarcomeres to contact or even encircle virtually every mitochondrion (Fig. 3) (Harrison and Roberts 2000). Tracheoles within flight muscles of flies and butterflies have been observed to fill with fluid at rest and air during activity (Wigglesworth and Lee 1982). This would indicate that gas exchange with mitochondria in active FM should occur through the gas phase. Moreover, tracheal O_2_ partial pressures of honeybees appear to be very high (8–10 kPa, 60–75 mmHg) (Wigglesworth and Lee 1982), relative to intracellular estimates of those in muscle of active humans (0.13–1.33 kPa, 1–10 mmHg) (Ortiz-Prado et al. 2019). With flight, these pressures also appear to remain constant suggesting that oxygen conductance is high and closely matches the need for O_2_ delivery during flight (Wigglesworth and Lee 1982). This presents an interesting challenge to design systems to measure mitochondrial function in a gaseous environment.

There are further considerations in the contexts of electron transport components. Two decades ago, estimates using spectroscopic methods were made to quantify cytochromes c, b, and aa3 in order to compare relative abundances of complexes III, IV and cytochrome c in honeybee FM. In comparison to rat liver, there were few differences, despite substantially lower OXP capacities of liver mitochondria (Suarez et al. 1996). The researchers contended that high flux honeybee FM mitochondria may have coordinated interactions among respiratory complexes, thereby increasing interactions. We now know that respiratory complexes arrange into supercomplexes, putatively to enhance reaction rates. Unexpectedly, work using blue native PAGE found little evidence for supercomplex formation in drosophila, yet there were elevated levels of ubiquinone and cytochrome c (Shimada et al. 2018), and this may enhance rates of electron transfer.

## A paradigm shift: are FM mitochondria highly connected*?*

Recent insights from skeletal muscle suggest an energy transmission system exists to promote ATP synthesis deep within myocytes (Glancy et al. 2015). Skeletal myocyte mitochondria of mammals form connected reticula (Picard et al. 2013), and these may be present in cardiomyocytes (Glancy et al. 2017; Ghosh et al. 2018) (Fig. 3). Spatially separated components of the OXP system occur within and among reticulated mitochondrial networks (Glancy et al. 2015).

Imaging methods have revealed that proton (H^+^) pumping respiratory complexes are most concentrated in rodent cardiomyocyte mitochondria immediately below the cell surface where O_2_ is richest (Fig. 3c). Given that H^+^s can be transferred an order of magnitude faster than ATP (Agmon 1995; Hubley et al. 1995, 1996), these ions may flow deeper and faster within myocytes via reticulated mitochondria (Glancy et al. 2017, 2015). These were theorised to power ATP synthesis in the core of myocytes (Glancy et al. 2015). Does such a reticulated mechanism occur in FM? If so, this would question the view of mammalian mitochondrial network roles in circumventing issues of oxygen delivery, because in FM trachea deliver O_2_ directly to mitochondria (Fig. 3d). Some have contended that the networking of mitochondria may also enhance ATP/ADP exchange in mammals and support cardiac function with diminished CK shuttles (Glancy et al. 2017, 2015). However, while H^+^-transport systems may support moderate activities in cardiac muscle, it remains to be determined if this system can meet the extreme adenylate transport/exchange rates required to operate FMs. Furthermore, adenylates must still diffuse through large FM fibrils to power contraction.

## A remarkable model

Over ~ 370 million years ago, insects evolved flight (Misof et al. 2014; Glancy et al. 2017). Intense selective pressures over the next 100 million years have since optimised FM metabolism and these compact motors for sustained power within ultimate athletes. On a whim, bees and wasps fire up FMs and oscillate loaded wings at frequencies equal or faster than those of the pistons in a “red-lined” Formula 1 race car motor (~ 12,000 rpm). Bees and other insects have achieved this feat in the face of apparent diffusion barriers for oxygen and adenylates. These molecules otherwise impede vertebrate muscles and when impaired manifest in myopathies such as heart disease. Resolving how these barriers have been overcome may reveal a means to better understand metabolic disease, or quite possibly unveil misconceptions of our present mammalian models. This could be particularly relevant to how mitochondrial populations interact and how electron transport systems operate at extreme rates and deal with oxidative stress. Exploring these animals will also further explain how some of the Biosphere’s fastest muscle types power flight.
